# Synergistic Interactions between *Drosophila* Orthologues of Genes Spanned by *De Novo* Human CNVs Support Multiple-Hit Models of Autism

**DOI:** 10.1371/journal.pgen.1004998

**Published:** 2015-03-27

**Authors:** Stuart J. Grice, Ji-Long Liu, Caleb Webber

**Affiliations:** Medical Research Council Functional Genomics Unit, Department of Physiology, Anatomy and Genetics, University of Oxford, Oxford, United Kingdom; The Hebrew University of Jerusalem, ISRAEL

## Abstract

Autism spectrum disorders (ASDs) are highly heritable and characterised by deficits in social interaction and communication, as well as restricted and repetitive behaviours. Although a number of highly penetrant ASD gene variants have been identified, there is growing evidence to support a causal role for combinatorial effects arising from the contributions of multiple loci. By examining synaptic and circadian neurological phenotypes resulting from the dosage variants of unique human:fly orthologues in *Drosophila*, we observe numerous synergistic interactions between pairs of informatically-identified candidate genes whose orthologues are jointly affected by large *de novo* copy number variants (CNVs). These CNVs were found in the genomes of individuals with autism, including a patient carrying a 22q11.2 deletion. We first demonstrate that dosage alterations of the unique *Drosophila* orthologues of candidate genes from *de novo* CNVs that harbour only a single candidate gene display neurological defects similar to those previously reported in *Drosophila* models of ASD-associated variants. We then considered pairwise dosage changes within the set of orthologues of candidate genes that were affected by the same single human *de novo* CNV. For three of four CNVs with complete orthologous relationships, we observed significant synergistic effects following the simultaneous dosage change of gene pairs drawn from a single CNV. The phenotypic variation observed at the *Drosophila* synapse that results from these interacting genetic variants supports a concordant phenotypic outcome across all interacting gene pairs following the direction of human gene copy number change. We observe both specificity and transitivity between interactors, both within and between CNV candidate gene sets, supporting shared and distinct genetic aetiologies. We then show that different interactions affect divergent synaptic processes, demonstrating distinct molecular aetiologies. Our study illustrates mechanisms through which synergistic effects resulting from large structural variation can contribute to human disease.

## Author Summary

Autism spectrum disorders (ASDs), which are characterised by poor social interaction and repetitive behaviours, are in part caused by genetic variation. A number of genes that vary in copy number in ASD patients have been identified, many of which were known to function at the neuronal synapse. We theorised that in some cases the dosage change of multiple genes simultaneously, rather than singularly, may lead to faulty neuronal development, and contribute to ASD. To test this, we asked whether alterations in these candidate genes would cause neuronal synapse and sleep/rest changes using the fruit fly *Drosophila*, and validated this model using single-gene models. We considered the simultaneous change of pairs of genes that were jointly affected by a large human copy number variant (CNVs), which are structural changes in the genome. In three of four CNVs, mutations in subsets of genes synergistically interacted to cause neuronal changes comparable to the single gene candidates. We also observed that the changes in synapse size followed the direction of the human gene copy number change. Finally, we show that different interactions affect the development of the synapse through different mechanisms, allowing us to identify distinct molecular alterations that illuminate the etiological heterogeneity of ASD.

## Introduction

Autism spectrum disorders (ASDs) comprise a large group of complex neurodevelopmental diseases that are influenced by genetic and environmental factors [1]. They are characterised by altered cognitive function including poor social and verbal interaction capability, and repetitive and stereotyped verbal and non-verbal behaviours [1]. ASDs are highly heritable (∼90% monozygotic twin studies); however, the genetic cause has been identified in less than 30% of cases, while the increase in risk between di-zygotic twins is comparable to that of first degree siblings [2], suggesting that ASD-causative alleles are likely to be both numerous and rare [3].

Recently, large numbers of autistic individuals, with unaffected family members, have been shown to possess *de novo* copy number variants (CNVs) [2,4–6]. In addition, many rare variant studies have identified pathways or processes that are commonly contributed to by significant proportions of those genes found to be disrupted [7–9]. Two additional striking findings from a recent study into the genes affected by 192 *de novo* CNVs identified in individuals with ASD have also been identified [9]. Firstly, many of these CNVs affect genes that appear to operate in the same functional pathway/network and, secondly, a significant proportion of individual CNVs (33%) simultaneously affect multiple genes whose proteins interact within that functional pathway [9]. This raises the possibility that it is the combined effect of these genes’ copy number change that causally contributes to these patients’ autistic phenotypes. Combinatorial effects have also been observed beyond *de novo* variants, where an increased risk of ASD resulting from multiple distinct and inherited CNVs has been reported [10]. However, while the contribution from combinatorial effects of genetic variation has been proposed by computational and statistical analyses, these hypotheses have yet to be validated *in vivo*. Here, we use *Drosophila* as an *in vivo* system to examine genetic interactions that may contribute to neurological phenotypes like ASD.

Understanding the interactions between genes implicated in autism requires a tractable, high-throughput *in vivo* system. This is particularly important as patient genotypes possess variants affecting many genes, thus generating an exponential number of potential interactions. To this end, the fruit fly *Drosophila melanogaster* offers a versatile tool in which neurodevelopment and behaviour can be studied in isogenised genetic backgrounds, and under controlled environmental conditions [11–13]. To detect single and combinatorial gene dosage effects in the fly, we examine two neurological phenotypes, namely (1) abnormalities in larval neuromuscular junction (NMJ) bouton number and (2) circadian defects apparent through abnormalities in adult sleep rest cycles. The NMJ offers a sensitive *in vivo* system to identify interactions that alter synaptic growth and maturation [14] and has proved a valuable tool for studying genes associated with neurodevelopmental disorders including autism spectrum disorders, intellectual disability and neuropsychiatric diseases [15–19]. For example, mutations in Neurexin IV, the *Drosophila* orthologue of the autism gene *CTNAP2*, have been shown to decrease NMJ bouton number and the abundance of glutamate receptors that oppose the active zones. Circadian rhythm activity defects have been previously reported in *Drosophila* neurodevelopmental models, including fragile X syndrome and Angelman syndrome, and can be an indicator and causative factor of neurodevelopmental and neurodegenerative disorders in humans [20–23]. Recent studies in *Drosophila* have also identified sleep abnormalities in mutations of the candidate ASD gene *cullin 3* (CUL3) [24–26]. Furthermore, sleep and circadian abnormalities are both significantly associated with ASD: Sleep disturbance is experienced by up to 80% of individuals with ASD, and while more strongly associated with ASD than other neurodevelopmental disorders it is not associated with intellectual disability, which is however frequently comorbid with ASD [22].

In this study, we modelled the effects of gene dosage changes on *Drosophila* neurological readouts using gene sets derived from multigenic *de novo* CNVs that had been identified in patients with autism [5,27–29]. We focussed our attention on the unique *Drosophila* orthologues of genes affected by these CNVs whose protein products had previously been found to participate in an ASD-associated interaction network, and which had a role in neural functioning [9]. To do this, we first considered those CNVs that changed only a single gene in the ASD-associated network, and show that the dosage alterations in the *Drosophila* orthologue yields neurological defects similar to those previously reported in *Drosophila* neurodevelopmental disease models [30,31]. We next looked at CNV gene sets that affected multiple genes in the ASD-associated network. Amongst these genes, no heterozygous mutation in a single gene led to significant synaptic defects in the fly. However, pairwise crosses between heterozygously-mutated genes yielded neurological defects comparable to the monogeneic models. We observe that (*i*) pairwise combinatorial dosage effects amongst these genes are not additive, but clearly synergistic, and (*ii*) that when the direction of copy change of the orthologues in individuals with ASD is considered, the observed effect at the *Drosophila* synapse supports a model of convergent phenotypic outcome between distinct synergistically-interacting gene pairs. No effects were observed among gene pairs that included neuronally-expressed *Drosophila* genes whose orthologues were affected by these CNVs but that were not part of the ASD-associated network. We show that the combinations of genes drawn from these CNVs that interact are specific, supporting distinct molecular aetiologies underlying ASD. We also show that these specific interactions affect different molecular processes at the *Drosophila* synapse, supporting the role of distinct molecular ASD related aetiologies. In total, we identified synergistically-interacting orthologous pairs among 3/4 of the CNVs considered, demonstrating novel synergistic interactions that may contribute to the aetiology of autism.

## Results

**Fig 1:**
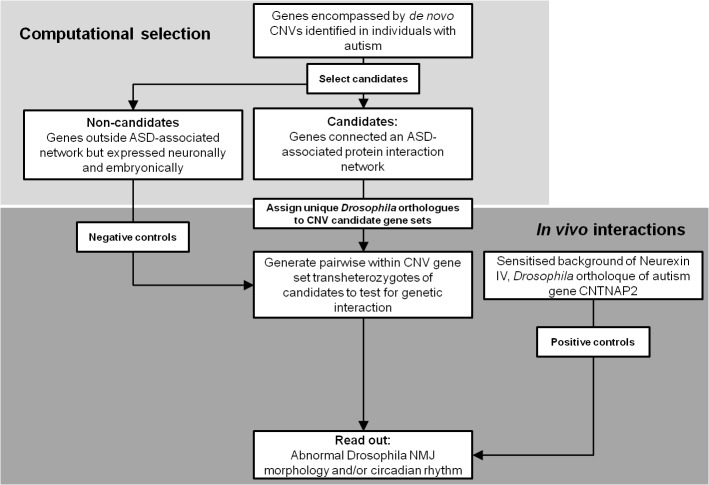
*Drosophila* screening strategy to detect interactions between the orthologues of genes simultaneously affected by a de novo CNVs identified in individuals with ASD (see Methods).

Previous studies applying network analyses to rare ASD associated genetic variants have proposed that these variants may genetically interact to exert their phenotypic influence in a patient [9,10]. To investigate the proposition of gene-gene interactions in these ASD cases, we used the fruit fly *Drosophila*. In particular, we modelled the effects of combinatorial heterozygous dosage changes of pairs of candidate genes, in the fly, and looked for synaptic and circadian defects. A schematic of our method is also set out in Fig. 1. Candidate genes were defined as those genes that had both (1) been identified to be previously affected in individuals with ASD by *de novo* CNVs, and additionally (2) those contributing to a large network of interacting proteins with roles in neural functioning, herein termed as an “ASD-associated network” [9]. Firstly, two CNVs were identified that affected only a single gene within the ASD-associated network: Specifically, 1 CNV affected *CTNND2* while another CNV affected *NOTCH1* (Table 1). These were brought forward as ‘monogenic’ candidates. Four additional *de novo* human CNVs were identified that each overlapped multiple ASD-associated network candidate genes, and where every candidate gene possessed a unique *Drosophila* orthologue. These CNVs gene sets were also taken forward for *in vivo* study (Table 1). In addition, from each of these 4 CNVs, two control genes were randomly selected and taken forward. These were genes that again possessed a unique *Drosophila* orthologue, and which were expressed in both the larval and adult nervous system (Table 1). Of the final 6 CNV gene sets taken forward for *in vivo* modelling, 2 sets were monogenic while 4 sets were polygenic. 4 were derived from copy number losses while 2 were derived from copy number gains. Table 1 details the CNVs, directionality, human genes and corresponding *Drosophila* ortholouges for all experiments.

**Table 1:** Human CNVs, candidate (bold) and control genes, and their respective *Drosophila* orthologues.

CNV Identity	Chr	Start	End	CNV Type	Human Candidate genes	Drosophila Orthologues of candidate genes	Figure
11079_chr3_loss_197208363_l	3	197208363	198838422	Loss	*Phosphate Cytidylyltransferase 1, Choline, Alpha (PCYT1A)*	*fsn*	3
					***Discs large 1 (DLG1)***	***Discs large (dlg)***	3
					***p21-activated kinase 2 (PAK2)***	***p21-activated kinases (Pak)***	3
					*LOC220729*	*CG5359*	3
12289_chr5_loss_11403621_l	5	11403621	11493124	Loss	***catenin delta 2 (CTNND2)***	***p10 catenin (p120ctn)***	2
1946_301_chr9_gain_138505259	9	138505259	139336068	Gain	***Notch1***	***Notch (N)***	2
12235_chr9_gain_129907917_l	9	129907917	130512360	Gain	***Dynamin-1 (DNM1)***	***dynamin/shibire (shib)***	5
					*Prostaglandin E Synthase 2 (PTGES2)*	*su(p)*	5
					*SWI5 Recombination Repair Homolog (SWI5)*	*CG14104*	5
					***alpha-Spectrin (SPTAN1)***	***alpha-Spectrin (a-spec)***	5
12239_chr22_loss_17249508_l	22	17249508	18693261	Loss	***T-box 1 (TBX1)***	***optomotor-blind-related-gene-1 (orbg-1)***	4
					***Guanine nucleotide-binding protein subunit beta-like protein 1 (GNB1L)***	***CG13192***	4
					*histone cell cycle regulator (HIRA)*	*hira*	4
					solute carrier family 25 (SLC25A1)	*sea*	4
					***Zinc finger, DHHC-type containing 8 (ZDHHC8)***	***CG34449***	4
					***George syndrome critical region gene 8 (DGCR8)***	***partner of drosha (pasha)***	4
					***Septin 5 (SEPT5)***	***Septin 4 (Sept4)***	4
12691.p1_chr16_loss_68529466_s	16	68529466	71494580	Loss	***VAC14***	***CG5608***	S1
					***PH domain and leucine rich repeat protein phosphatase-like (PHLPPL)***	***PH domain leucine-rich repeat protein phosphatase***	S1
					***Splicing factor 3B subunit 3 (SF3B3)***	***CG13900***	S1
					***Calbindin 2 (CALB2)***	***Calbindin 53E (cbn)***	S1
					*tyrosine aminotransferase (TAT)*	*CG5608*	S1
					*C-type lectin domain family 18, member C (CLEC18C)*	*CG3626*	S1
					***AP1G1 adaptor-related protein complex 1, gamma 1 subunit (AP1G1)***	***AP-1γ***	S1
					***Dihydroorotate dehydrogenase (DHODH)***	***Dihydroorotate dehydrogenase (dhod)***	S1

CNVs were selected from previous studies that identified *de novo* or rare CNVs in the genomes of individuals with ASD (see Methods). CNVs post-fixed “_1” were taken from the study by Levy *et al*. [28], CNVs post-fixed “_s” were taken from the study by Sanders *et al*.[29], while the CNV 1946_301_chr9_gain_138505259 was taken from the AGP study [5]. The candidate and control genes were among those genes affected by the given CNV: The protein products of the candidate genes interacted in a previously identified network of interacting proteins associated with neural functioning, while the control genes’ protein products are expressed embryonically and neuronally.

### Modelling ASD genes in the fly with NMJ and circadian phenotypes

Singular and combinatorial effects resulting from the simultaneous dosage change of ASD-candidate genes were investigated by identifying changes in neuromuscular junction (NMJ) bouton number, and circadian rhythms (specifically alterations in the light/dark bias towards sleep and rest). As a complex disease with behavioural deficits relating to alterations in the human brain, ASD may not be wholly modelled in *Drosophila*. However, by enabling the rapid screening of multiple target genes the fly is a powerful model to test gene-gene interactions *in vivo*. It thus offers a tractable method to better understand the gene-gene interactions postulated to occur as a result from these large *de novo* CNVs. We believed bouton number, and circadian rhythms to be relevant because phenotypes because: (*i*) The fly NMJ, a tractable and highly characterised glutamatergic synapse, has been successfully used to detect synaptic defects in models of ASD, neuropsychiatric disease and intellectual disability [15,31]; (*ii*) circadian rhythm defects are associated with ASD and several fly ASD models [21,32,33].

### *Drosophila* models of monogenic forms of ASD yield neurological phenotypes

**Fig 2:**
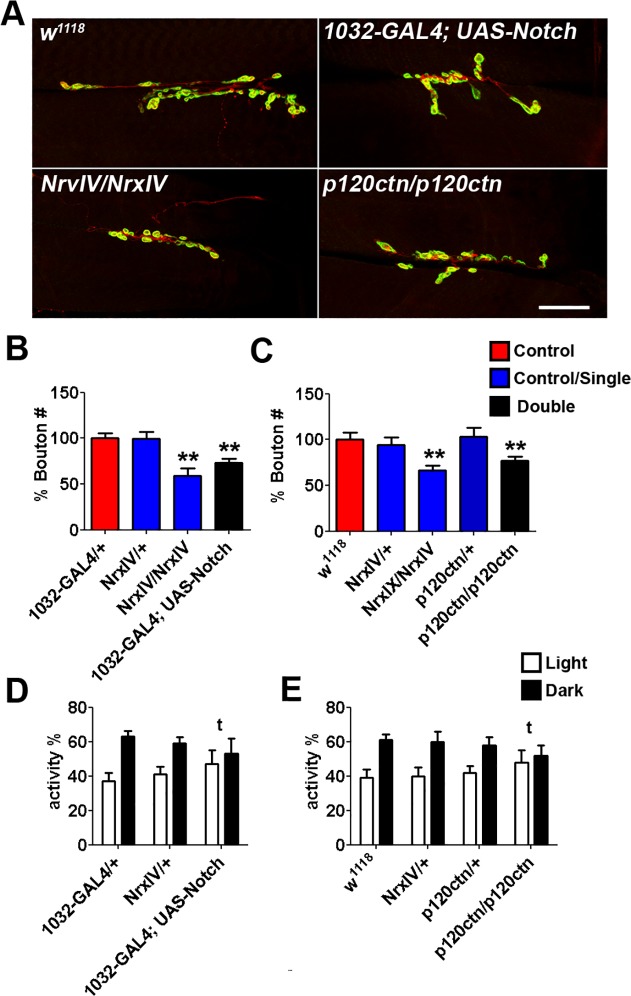
Bouton number at the *Drosophila* NMJ following the overexpression and mutation of the *Drosophila* unique orthologues of candidate genes identified from human autism-associated copy number variants (CNVs). **A.** Representative pictures of NMJs from *NrxIV/NrxIV* (using *Nrx-IV* ^*4304*^), Notch overexpessing (1032-GAL4, UAS-Notch), and *p120ctn/p120ctn* (using *p120ctn* ^*308*^) 3^rd^ instar larvae; Scale bar = 20μm. **B.** Homozygous disruption of *NrxIV*, the orthologue of the autism gene *CTNAP2*, provided a positive control and displayed a reduced NMJ bouton number as described previously. Heterozygous mutants of *NrxIV* yielded no bouton number reduction. Overexpression of *Notch* (1032-GAL4; UAS-Notch-full), whose human orthologue is duplicated in the *de novo* gain CNV_946_301_chr9_gain_138505259 (Table 1), gave reduced bouton numbers (n>20, Kruskal-Wallis test, ** P<0.01). **C.** Homozygous disruption of *p120ctn*, that is affected by the *de novo* loss CNVs 12289_chr5_loss_1140362 (Table 1), yields reduced bouton numbers. (n>20, Kruskal-Wallis test, ** P<0.01). Heterozygous mutants of *p120ctn* have no significant change on NMJ morphology. **D. and E.** Circadian sleep/rest rhythm analysis of candidate genes from the CNV sets. 1032-GAL4, UAS-Notch, and *p120ctn/p120ctn* flies lost the dark bias, displaying no significant difference between light/dark sleeping patterns (**t,** representing the crosses where no light/dark sleep/rest bias was observed). Light/dark sleeping bias was measured using student’s t-tests.

Two of the six CNV gene sets considered contained only one candidate gene. One monogenic gene set is derived from a loss CNVs that affected the orthologue of the *Drosophila* gene p120 catenin (*p120ctn*) with roles in cell adhesion and signal transduction, while the other CNV contained the evolutionarily conserved signalling molecule Notch, whose human orthologue was found to be copy number increased (Table 1). Mutants for neurexin IV (using *Nrx-IV* ^*4304*^), the orthologue of the autism gene *CNTNAP2*, and *w* ^*1118*^ were used as positive and wild type controls, respectively. All *Drosophila* stocks were isogenised to the *w* ^*1118*^ wild type background for 7 generations for this study. As previously described, we found that *NrxIV* homozygous null mutants display reduced bouton numbers, while heterozygote nulls have no observable difference when compared to wild type (Fig. 2A) [34].

The first monogenic CNV gene set we analysed was *Drosophila* Notch, the orthologue of human Notch1, derived from a human Chromosome 9 copy number gain CNV (Table 1). To investigate the increased expression of *Drosophila* Notch, we overexpressed *Drosophila* Notch (using UAS-Notch-Full) driven by the ubiquitous expression GAL4 driver 1032-Gal4 (Fig. 2B). While larvae overexpressing Notch had no overt effect on early larval survival, we observed reduced NMJ bouton numbers (n>20, Kruskal-Wallis test, ** P<0.01; Fig. 2B) showing that dosage increase in this gene yields synaptic phenotypes in *Drosophila*.

Next, we considered the monogenic CNV gene set corresponding to the loss of the *Drosophila* orthologue *p120ctn*. The previously described null mutant *p120ctn* ^*308*^ was isogenised to analyse hemizygous *p120ctn* loss [35]. However, *p120ctn* heterozygous null mutants displayed no significant change in NMJ (Fig. 2C) although homozygous p120ctn null mutants were found to display a significantly reduced bouton number (n>20, Kruskal-Wallis test, ** P<0.01, Fig. 2C). We note that, unlike in vertebrates, *Drosophila p120ctn* homozygous null mutants are viable [35].

We next looked for circadian rhythm defects in the monogenic CNV gene set orthologues *Notch* and *p120ctn* mutants. *Notch* overexpression larvae were reared at 16°C, and were transferred to 25°C during pupation, so to mitigate gross developmental defects. We analysed sleep/rest periods (measured as a contiguous 5 minute periods of inactivity) as a surrogate for looking at gross defects in rhythmicity. While all negative control and single mutants displayed normal light/dark differences in sleeping patterns (i.e more sleep/rest periods during the dark 12hrs; Fig. 2D, E), both *p120ctn* homozygous nulls (Fig. 2E) and the *Notch* (Fig. 2D) overexpressing flies all lost the dark bias and displayed no significant difference between light/dark sleeping patterns.

Taking these monogenic models together, we show that dosage change in *Drosophila* of the orthologues of known ASD diseases genes (*NrxIV*), and of ASD-candidate genes subject to *de novo* copy number increase (*notch*) and decrease (*P120ctn*) in human, all yield abnormalities at the NMJ, and in circadian rhythms (*notch* and *p120ctn*) (Fig. 2). We also find that despite differences in the direction of dosage change in *Drosophila* that are consistent with the copy change observed for these 3 genes in individuals with ASD, the bouton count at the NMJ is reduced in all models, supporting a convergent phenotypic outcome in both *Drosophila* and human.

### *Drosophila* models of polygenic causes of ASD are driven by genetic interactions

We next considered the four CNVs that each affected multiple genes within the ASD-associated network. For each, we asked whether the dosage change of their *Drosophila* orthologues singularly or in pairwise combination yielded NMJ synaptic or circadian abnormalities. The number of ASD-associated network candidate genes in each of the five CNVs with multiple candidate genes ranged from 2–6, with a mean of ∼4. The four CNVs consisted of three loss CNVs (11079_chr3_loss_197208363_l with two candidate genes; 12239_chr22_loss_17249508_l with five candidates; 12691.p1_chr16_loss_68529466_s with six candidates) and one gain CNVs (12235_chr9_gain_129907917_l with two candidates) (Table 1)

**Fig 3:**
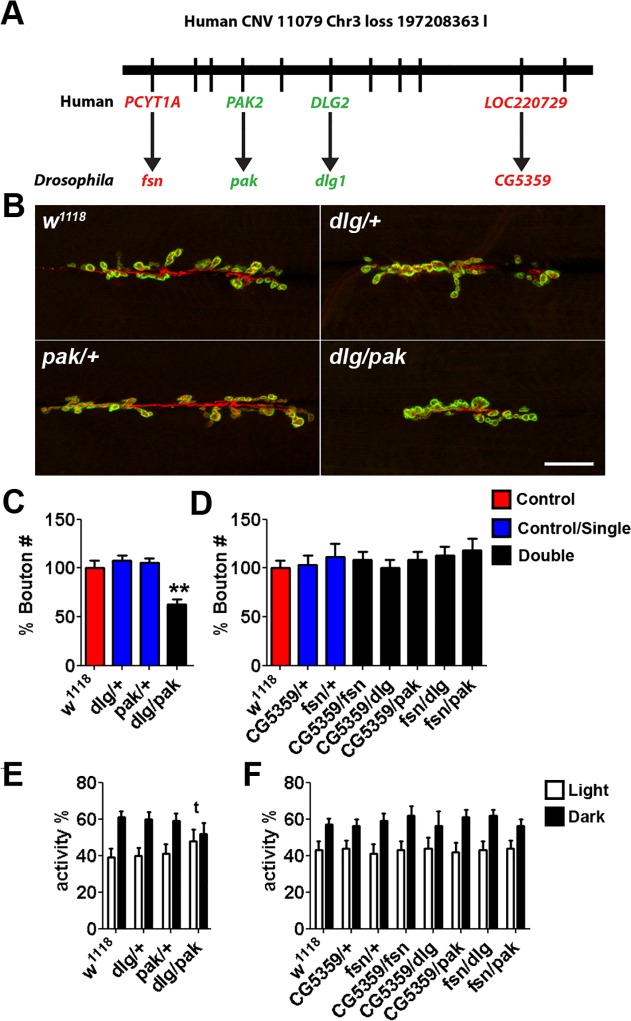
Synergistic interaction in *Drosophila* between *Dlg* and *Pak*, the orthologues of ASD-candidate genes from a *de novo* loss CNV 11079_chr3_197208363. **A.** The Locus of the CNV with mapped *Drosophila* orthologues (Candidates, green; controls, red). **B.** Representative pictures of NMJs from *dlg*/+ (using *dlg* ^*1*^), *pak*/+ (using *pak* ^*6*^), and *dlg/pak* 3^rd^ instar larvae; Scale bar = 20μm. **C.** Synaptic alterations were characterised by NMJ bouton number. Individual heterozygous mutants of candidate gene orthologues *dlg* and *pak* (*dlg/+* and *pak/+)* gave no significant change in NMJ morphology over *w* ^*1118*^ controls. However, *dlg*/*pak* transheterozygotes have reduced bouton numbers. (n>20, Kruskal-Wallis test, ** P<0.01). **D.** Non-candidate gene controls *fsn* (using *Fsn* ^*KG08128*^) and CG5359 (using *CG5359* ^*e03976*^) selected from genes found within CNV gave no significant NMJ phenotype singularly or when crossed to form transheterozygotes with *dlg* or *pak*. **E.** and **F.** Circadian rhythm analysis of candidate genes. All negative control **F.** and single mutants displayed normal light/dark differences in sleeping patterns. However, transheterozygote *dlg*/*pak* flies lost the dark bias, and displayed no significant difference between light/dark sleeping patterns (**t**).

The first multiple candidate gene CNV studied, human *de novo* loss CNV 11079_chr3_197208363 (Fig. 3A), contained two candidates: the septate junction protein discs large (*dlg*) and p21-activated kinase (*pak*), a serine/threonine-protein kinase [36], which has been previously shown to control the synaptic Dlg localisation. Isogenised transheterozygotes of the mutants *dlg* (*dlg* ^*1*^) and *pak* (*pak* ^*6*^) were used and bouton number analysed for synaptic alterations. Single *dlg* and *pak* heterozygous mutants alone displayed no significant change in NMJ morphology when compared to controls (Fig. 3B, C), whilst homozygous mutants are lethal, as previously reported [37,38]. However, *dlg/pak* transheterozygotes (although the correct full geneotype is *w* ^*1118*^, *dlg* ^*1*^; *+/+; pak* ^*6*^/*+* for this example, all transheterozygtes will be represented in the ‘*gene/gene*’ format going forward, for simplicity) displayed significant bouton number reductions (n>20 Kruskal-Wallis test, ** P<0.01; Fig. 3C). For additional controls, *Fsn* (using *Fsn* ^*KG08128*^) and CG5359 (using *CG5359* ^*e03976*^), which were selected from genes found within CNV 11079_chr3_197208363 but did not participate in the ASD-associated network, were crossed to *dlg* and *pak* heterozygotes but no significant NMJ morphology changes were observed (Fig. 3D). To look for circadian behavioural phenotypes, day/night sleep patterns of adult flies were again analysed. Wild type flies and all negative controls (transheterozygote crosses with *Fsn* ^*KG08128*^ and *CG5359* ^*e03976*^; Fig. 3E, F) and single mutants displayed normal light/dark differences in sleeping patterns, with more sleeping periods in the dark. However, *dlg/pak* flies lost the dark bias (Fig. 3E), displaying no significant difference between light/dark sleeping patterns. Thus, *dlg/pak* flies demonstrated synergistic effects, displaying both reduced NMJ bouton number and circadian rhythm defects only in the transheterozygotes.

**Fig 4:**
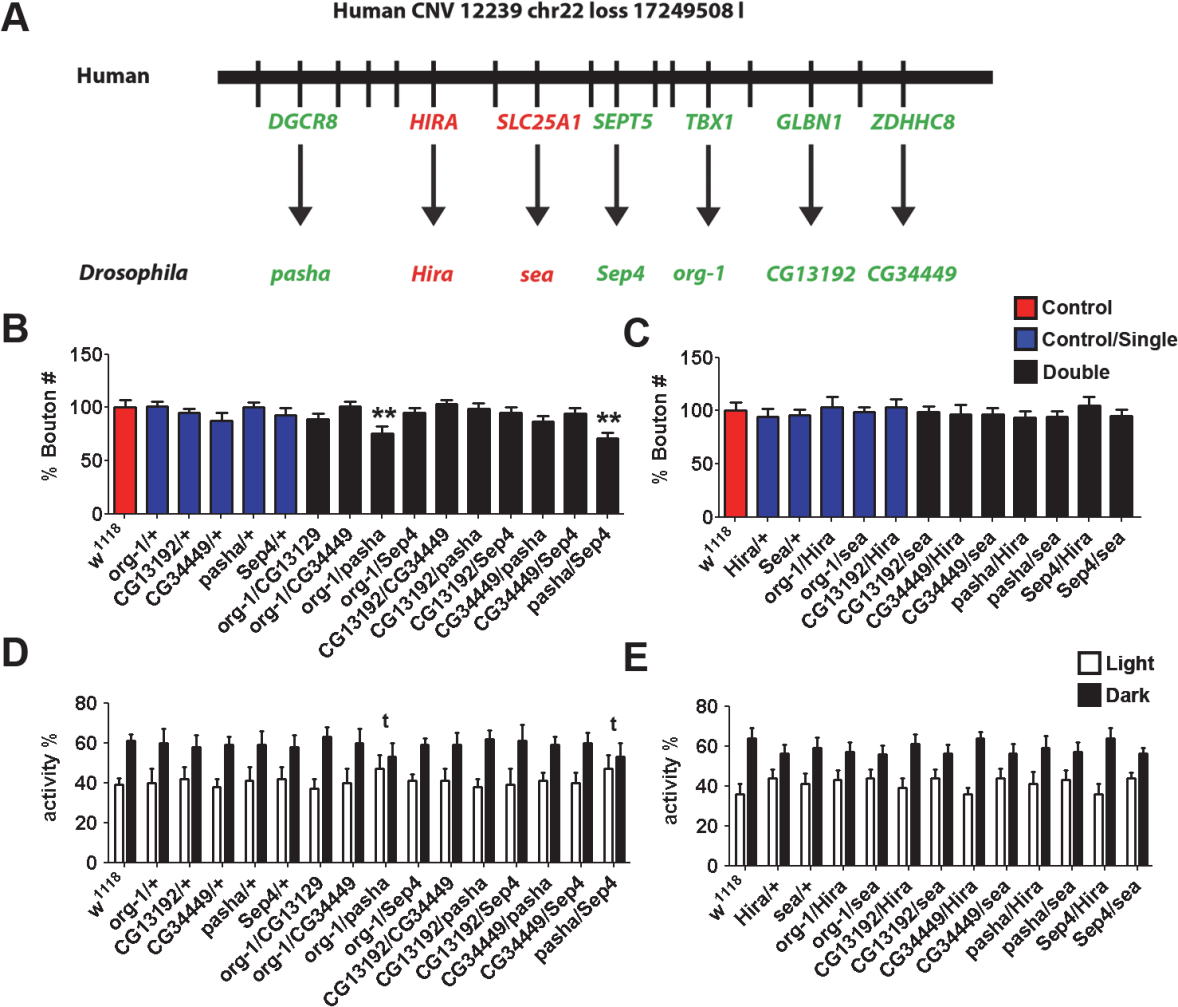
Synergistic interactions in *Drosophila* between *org-1, pasha* and *Sept4*, the orthologues of ASD-candidate genes from a *de novo* loss CNV 12239_chr22_loss_17249508_l. **A.** The Locus of the CNV with mapped *Drosophila* orthologues (candidates, green; control, red). **B.** Synaptic alterations were characterised by NMJ bouton number. Individual heterozygous mutants of 5 candidate gene orthologues (*org-1, CG13192, CG34449, pasha, Sep4*; blue bars) gave no significant change in NMJ morphology over *w* ^*1118*^ controls (red bar). *org-1*/*pasha* and *pasha*/*Sep4* transheterozygotes display reduced bouton. (n>20, Kruskal-Wallis test, ** P<0.01). The mutants *pasha* ^*LL03360*^, org-1^MB01466^ and *Sep4* ^*NP7170*^ were used respectively. **C.** Non-candidate gene controls *Hira* (using *Hira* ^*185*^) and *Sea* (using *sea* ^*EP3364*^) selected from genes found within CNV gave no significant NMJ phenotype singularly or when crossed to form transheterozygotes with candidate genes. **D.** and **E.** Circadian rhythm analysis of candidate genes. All negative control and single mutants displayed normal light/dark differences in sleeping patterns. However, *org-1*/*pasha* and *pasha*/*Sep4* transheterozygotes lost the dark bias, and displayed no significant difference between light/dark sleeping patterns (**t**).

Analysis of a second human *de novo* loss (12239_chr22_loss_17249508_l; Table 1; Fig. 4A), covering the recurrent 22q11.2 microduplication critical region [39], found no evidence of abnormalities in NMJ bouton count nor circadian cycle in the single heterozygote mutants of any of the 7 genes examined (5 candidates and 2 controls; Fig. 4). However, the two transheterozygous combinations of *partner of drosha* (*pasha*; using *pasha* ^*LL03360*^) [40] with *optomotor-blind-related-gene-1* (*org-1*, using org-1^MB01466^) [41] and that of *pasha* with Septin4 (*Sep4*, using *Sep4* ^*NP7170*^) were both found to have reduced bouton numbers (Fig. 4B; n>20, Kruskal-Wallis test, ** P<0.01, *p<0.05). These relations, however, were not transitive as the combination of org-1 and *Sep4* (*org-1/Sep4*) did not yield these phenotypes. Similarly, only the org-1/*pasha* and *pasha*/*Sep4* transheterozygote flies also lost the dark bias, displaying no significant difference between light/dark sleeping patterns while org-1/*Sep4* did not (Fig. 4D). No significant NMJ morphology or sleep/rest changes were seen when negative controls hira (using *hira* ^*185b*^) and *sea* (using *sea* ^*EP3364*^) were crossed to form transheterozygotes with the candidates (Fig. 4C, E). Thus, again, we observe synergistic combinatorial effects, with both NMJ bouton number and circadian rhythm defects apparent only in the transheterozygotes for this second multigenic loss CNV gene set. However, a final multigenic loss CNV gene set, 12691.p1_chr16_loss_68529466_s, failed to yield any significant NMJ bouton number or circadian defects amongst single or pairwise heterozygotes (Table 1; S1 Fig.).

### A *Drosophila* model of a human *gain* CNVs supports convergent aetiologies following copy number change in ASD

**Fig 5:**
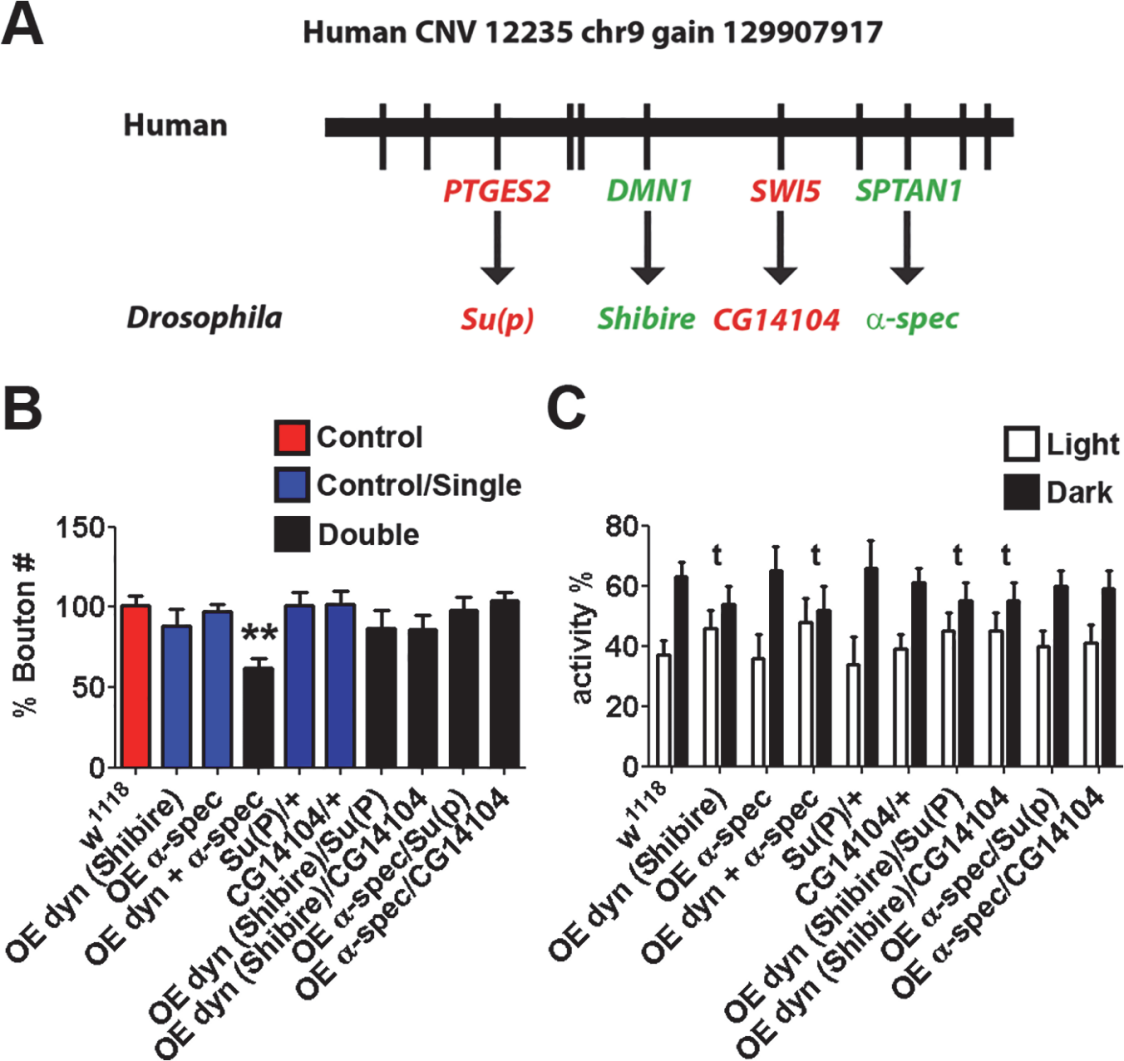
Synergistic interactions in *Drosophila* between *shibire* and *alpha spectrin*, the orthologues of ASD-candidate genes from a *de novo* gain CNV 12235_chr9_gain_129907917_l. **A.** The Locus of the CNV with mapped *Drosophila* orthologues (Target, green; control, red). **B.** Synaptic alterations were characterised by NMJ type IB bouton number. Individual heterozygous mutants of candidate gene orthologues gave no significant change in NMJ morphology over *w* ^*1118*^ controls. However, *Shibire* and *alpha-spectrin* double over expressers display reduced bouton numbers (using 1032-GAL4; UAS-Dynamin/UAS-alpha-spectrin; n>20, Kruskal-Wallis test, * P<0.05). Non-candidate gene controls *Su(P)* (using *Su(P)* ^*EY13245*^) and *CG14104* (using *CG14104* ^*f07593*^) selected from genes found within CNV gave no significant NMJ phenotype singularly or when crossed to form transheterozygotes with candidate genes. **C.** Circadian rhythm analysis of candidate genes. Negative controls and candidate gene orthologue overexpression of *alpha-spectrin* displayed normal light/dark differences in sleeping patterns singularly or when crossed. However, *Shibire* overexpression, and co-overexpression with *alpha-spectrin* lost the dark bias, and displayed no significant difference between light/dark sleeping patterns (**t**).

We next analysed a gene set derived from a copy number gain (12235_chr9_gain_129907917_l, Fig. 5A), by generating constructs for overexpression, and by employing the UAS-GAL4 over-expression system. The two ASD-associated network genes, dynamin (*Shibire*) and alpha spectrin, when over-expressed together display a decreased NMJ bouton number (Fig. 5B) and lost the dark bias to sleep (Fig. 5C). The observed decrease in bouton number following pairwise over-expression of these candidate genes duplicated in humans with ASD is consistent with the bouton number decrease also observed among the pairwise disruptions of candidate genes found to be deleted in humans with ASD. Although the dynamin (*Shibire*) over-expresser alone also showed a loss of dark sleep bias in this case, individually-driven genes displayed no significant change in NMJ morphology over *w* ^*1118*^ controls. No significant NMJ morphology changes are seen when non-ASD-network controls from the CNV gene set Su(P) (using *Su(P)* ^*EY13245*^) and CG14104 (using *CG14104* ^*f07593*^) are crossed into the overexpressing backgrounds (Fig. 5B for NMJ analysis and Fig. 5C for sleep/rest analysis).

Taking all the polygenic models together, with one exception (dynamin (*Shibire*) dark bias; Fig. 5B), we show that only particular pairwise combinations of dosage change generate interactions that yield neurological phenotypes comparable to those observed in the monogenic models (Figs. 2–5). As with the mongenic CNV gene sets examined, among the 3 CNV gene sets that demonstrate pairwise interactions, we observe directionality effects in NMJ bouton count that are consistent with a convergent phenotypic outcome. Finally, singularly or in pairwise combinations, we observed no phenotypes for any model involving non-ASD-associated network genes.

### Specific subsets of the candidates modify the Neurxin IV background

**Fig 6:**
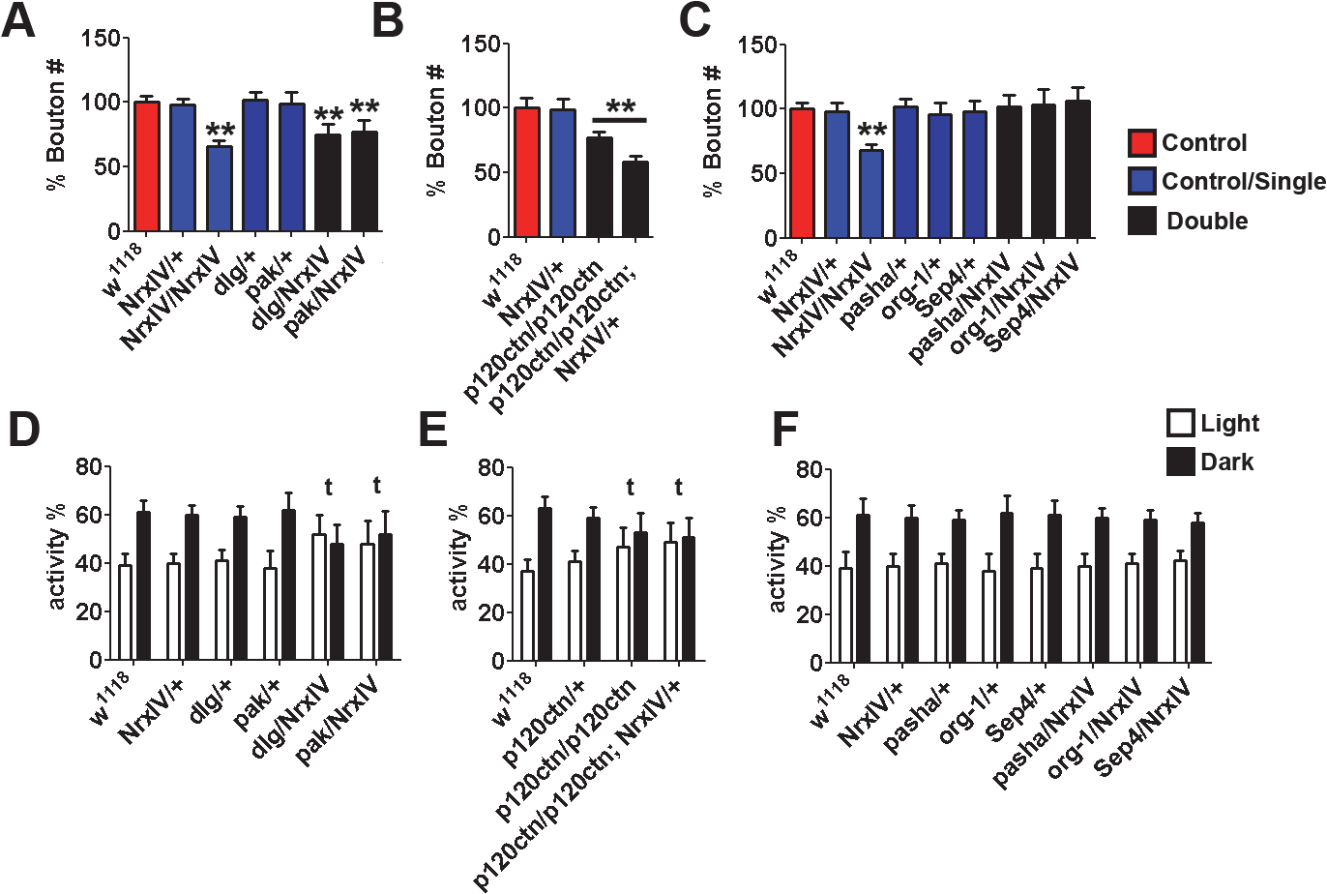
Selective genetic interactions observed between the *Drosophila* orthologues of ASD candidate genes and Neurexin IV. A sensitised background of Neurexin IV (*NrxIV/+*), the orthologue of the autism gene *CTNAP2*, was used to look for interactions between *NrxIV* and the ASD candidate gene orthologues *dlg, pak, p120ctn, pasha*, and *org-1*. **A**. *dlg/+, pak/+* and *NrxIV/+* heterozygous mutants have no significant change in NMJ morphology over *w* ^*1118*^ controls. However, *dlg*/*NrxIV* and *pak*/*NrxIV* crosses both displayed reduced bouton numbers (n>20 Kruskal-Wallis test, ** P<0.01). **B.** Significant NMJ morphology changes are also seen for the *NrxIV* cross with the homozygous *p120ctn* mutant cross, when compared to the homozygous *p120ctn* mutant cross alone (n>20 Kruskal-Wallis test, ** P<0.01). **C.** No NMJ morphology changes were observed when *pasha* and *org-1*, both from human *de novo* loss 12239_chr22_loss_17249508_l, were crossed to *NrxIV/+*. **D. to F.** Circadian rhythm analyses of models in Panels A, B and C support observed genetic interactions: d*lg*/*NrxIV, pak*/*NrxIV* transheterozygotes and the *NrxIV* cross with the homozygous *p120ctn* mutant flies lost the dark bias, and displayed no significant difference between light/dark sleeping patterns (**t**), while *pasha*/*NrxIV* and *org-1*/*NrxIV* transheterozygotes displayed no abnormal circadian phenotype.

Understanding the functional relationships between genes underlying ASD will help elucidate the processes that lead to neurological dysfunction and ultimately may pinpoint common mechanisms that lead to the disorder. To test the relationship between our candidate genes and a known ASD candidate we crossed subsets of our candidates with neurexin IV, the orthologue of the autism gene *CNTNAP2*. From our candidate list we selected *dlg, pak* and *p120ctn* which have functional roles in cell adhesion processes that may involve *neurexin IV* [35]. We crossed heterozygous *dlg* (*dlg* ^*1*^), *pak* (*pak* ^*6*^) and *p120ctn* homozygotes (*p120ctn* ^*308*^/*p120ctn* ^*308*^) to a sensitised background of *NrxIV* (*Nrx-IV* ^*4304*^/*+*) and analysed bouton number. In all three cases, the transheterozgotes of each of *dlg, pak* and *p120ctn/p120ctn* in combination with *NrxIV* (*Nrx-IV* ^*4304*^/*+*) synergistically yielded reduced bouton number and displayed a loss in the dark bias to sleep suggesting that these proteins may act in the same pathway (Fig. 6A, B for NMJ analysis and Fig. 6D, E for sleep/rest analysis). It is worth noting that *p120ctn/p120ctn* flies in combination with *NrxIV* had a significantly reduced survival.

We next crossed *pasha, sep4*, and org-1 heteozygotes with *neurexin IV* to see if modification of the NMJ and dark sleep bias was a common feature when alleles were present in the sensitised *NrxIV* (*Nrx-IV* ^*4304*^/*+*) background. In these cases no significant changes to the NMJ phenotypes (Fig. 6C) or sleep/rest rhythms (Fig. 6F) were observed suggesting that *pasha, Sep4* and org-1 are acting on non-converging pathways.

### Genetic interactions subsets cause differential synaptic defects

**Fig 7:**
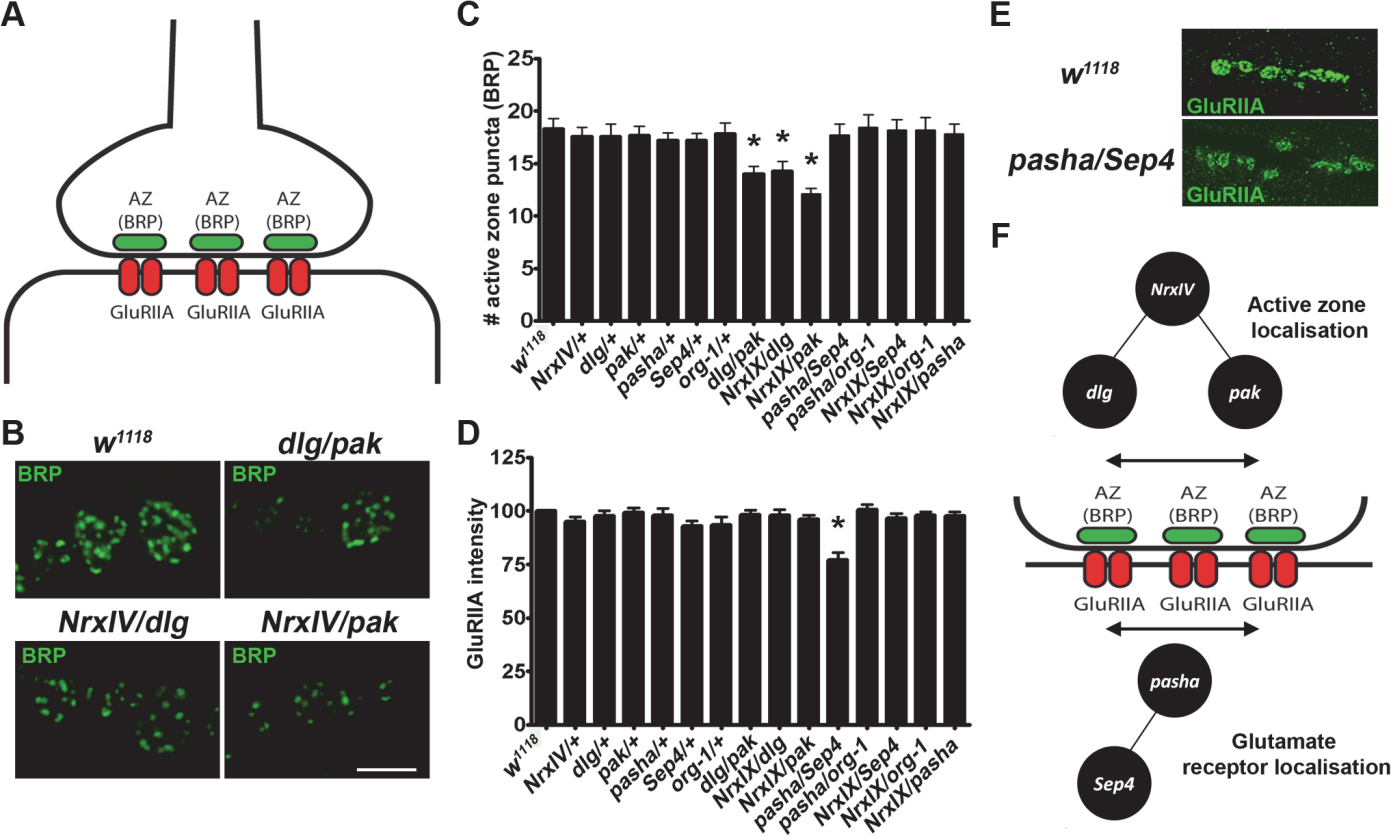
Different genetic interactions effect distinct synaptic defects suggesting that distinct molecular aetiologies underlie ASD. **A.** The *Drosophila* NMJ contains presynaptic active zones (labelled by Bruchpilot, BRP) and postsynaptic glutamate receptor (labelled by GluRIIA). **B.** Representative images of BRP staining demonstrate a reduced number of active zone (BRP) puncta in the transheterozygotes *dlg/pak, NrxIV/dlg* and *NrxIV/pak* as compared to control (*w* ^*1118*^), **C.** The number of active zone (BRP) puncta (normalised to bouton size) were significantly reduced in *dlg/pak, NrxIV/Dlg* and *NrxIV/Pak* transheterozygotes. **D.** The fluorescence of the post-synaptic glutamate receptors were scored and normalised to bouton size HRP levels. *Pasha/Sep4* transheterozygotes were the only genotype to demonstrate decreased glutamate receptor abundance. **E.** Representative images of GluRIIA staining demonstrating the reduced fluorescence in the transheterozygote *Pasha/Sep4* when compared to control. **F.** A schematic showing the sub-types of genetic interactions supporting distinct molecular aetiologies underlying ASD that converge to yield defects at the synapse.

To better understand how these interacting and non-interacting gene pairs exert common circadian and synaptic phenotypic effects, we next looked for molecular defects at the synapse. Single homozygous mutations of *Drosophila* ASD gene orthologues display defects in synapse development [18,42]. Examples of these defects include alterations in glutamate receptors abundances, active zone numbers, and presynaptic and postsynaptic structural defects at the larval NMJ [15,17,18,21,30,42]. To investigate whether gene dosage changes from the transheterozygote subsets above cause molecular synaptic defects, we looked for alterations in active zone localisation and glutamate receptors abundance at the *Drosophila* larval NMJ. *Drosophila* active zones are identified by staining with the protein bruchpilot (BRP, Fig. 7A), which is positioned presynaptically and opposite to the postsynaptic neurotransmitter receptors. We measured BRP foci and normalised them to bouton area from transheterozygotes of *NrxIV, dlg, pak, pasha, Sep4 and org-1*. We found that all transheterozygous crosses between nrxIV, *dlg* and *pak*, which we have shown to genetically interact, displayed a reduction in BRP localisation at the synapse (Fig. 7B, C). However, this was not observed for the genetically-interacting transheterozygotes *pasha/Sep4* or *pasha/org-1* or single heterozygous mutants and controls. We next explored whether dosage changes in our candidate genes might lead to the destabilisation of the clustering of the postsynaptic glutamate receptors, by studying the levels of GluRIIA at the synapse. Again, alterations in glutamate receptor subtypes have been discovered in single homozygous mutations of *Drosophila* ASD gene orthologues [42,43]. In this case, we found that out of all single and transheterozygote crosses, only *pasha/Sep4* displayed a reduction in the levels of GluRIIA (Fig. 7D, E). Taken together, our findings demonstrate that distinct molecular developmental alterations are associated with the different genetically interacting gene combinations, supporting the idea that distinct molecular aetiologies may contribute to ASD by converging on common phenotypic outcomes (Fig. 7F).

## Discussion

In this study we have developed an *in vivo* model system in *Drosophila* to determine how genes can synergistically interact within ASD associated *de novo* CNVs. Specifically, we have shown that (*i*) of the 4 human CNVs containing 2, 2, 5 and 6 network-identified candidate genes respectively (from a combined total of 114 copy-changed protein-coding genes), pairwise interactions between *Drosophila* orthologues yielding changes in the neuromuscular junction (NMJ) bouton number and circadian rhythms were observed for 3 CNVs; (*ii*) that the interactions observed are synergistic, as opposed to additive, in nature, and (*iii*) that the synaptic bouton counts observed following the simultaneous dosage change of all 5 pairs of interacting CNV candidate genes’ orthologues within *Drosophila* support a convergent phenotypic outcome arising from these genes’ dosage change for the individuals with ASD within whom they were identified (Figs. 2–5, S2). We show that the combinations of genes drawn from these CNVs that interact are specific, both within a CNV (Fig. 4) and between CNVs (Fig. 6), supporting distinct aetiologies underlying ASD. Finally, we go on to show these specific interactions act through different molecular aetiologies, supporting the role of distinct molecular aetiologies in ASD (Fig. 7).

The synergistic, as opposed to additive, nature of the pairwise genetic interactions that we observe in *Drosophila* has important consequences for identifying the genetic causes of ASD, and (*i*) the conserved orthology of the interactors, (*ii*) the human orthologues’ participation in an ASD-relevant network constructed from known mammalian interactions, and (*iii*) the concordance between the direction of dosage change and phenotype all support the inter-species relevance of our findings. Although there are over 100 ASD candidate genes currently identified, at least 70% of the genetic causes remain to be explained [9,44]. The presence of multiple genetic variants in many patients [29,45] suggests that inherited variants might lead to ASD through the combinatorial effects of distinct deleterious variants which affect a shared biological pathway (Fig. 6) [10]. Where variants that act additively to cause ASD in a proband are inherited from each parent, those variants individually may cause detectable ASD-relevant traits in the parents [10,46,47]. However, if combinations of variants act only synergistically to cause ASD, there would be no expectation of ASD-relevant traits in either parent. Importantly, if sub-threshold ASD traits affect fecundity then variants that are only deleterious in combination may rise to a higher frequency in the population. Our results in *Drosophila* show that only particular combinations of dosage variants act together to yield an abnormal phenotype (Fig. 4 and Fig. 6). Identifying those variants that contribute to ASD only in combination with other specific variants, amongst a background of large amounts of non-contributing genetic variation, will be challenging because the variety of gene variant combinations is extremely large, and allele frequencies are likely very rare.

The genes participating in the pairwise genetic interactions identified by our screen were *discs large (dlg*: human orthologue (h.o) *DLG1), p21-activated kinase (pak*: h.o. *PAK2), p20 catenin (p120ctn*: h.o. *CTNND2), Notch (N*: h.o Notch 1), *shibire/dynamin (shi/dynamin*: h.o. *DNM1), alpha-Spectrin (α-spec*: h.o. *SPTAN1), optomotor-blind-related-gene-1 (org-1*: h.o. *TBX1), partner of drosha (pasha*: h.o. *DGCR8)* and *Septin* 4 *(Sep4*: h.o. *SEPT5)*. An examination of CNVs listed in the Database of Genomic Variants (DGV) [48] reveals that most of these genes are found to be individually dosage changed in the same direction in apparently healthy individuals (DLG1, 7 CNVs; PAK2, 1 CNV; DNM1, 1 CNV; SPTAN, 1 CNV; SEPT5, 2 CNVs; TBX1, 9 CNVs; DGCR8 5 CNVs). However, only one of these CNVs might simultaneously change two genes that our study demonstrate genetically-interact in the fly (variant nsv828939; [49]) and CNVs strongly implicated in ASD have previously been reported in apparently healthy individuals [47,50]

Many of the interacting genes have known functions in the nervous system. For example the localisation of the septate junction and neuronal adhesion protein Dlg at the NMJ has been shown to be regulated by Pak serine/threonine-protein kinase activity [36]. In addition, it is interesting to point out that p21-activated kinase (PAK) has been shown to interact with the protein SHANK3 in rat, whose disruption can also cause ASD, with mutant *Shank3* altering actin dynamics driven by PAK signalling [51]. Destabilisation of the actin filaments at the NMJ leads to defective NMDAR-mediated synaptic current in neurons. PAK inhibitors have also been shown to rescue fragile X syndrome phenotypes in Fmr1 KO mice [52], suggesting an important role for Pak serine/threonine-protein kinase activity in ASD and ID. The gene *alpha-spectrin*, which we show genetically interacts with the dynamin protein shabire [53], is known to cross link actin, and has been shown to be important for the localisation of Dlg at the synapse [54]. The phenotypes resulting from the combination of these genes’ variants suggests an important role for the control of synapse integrity via actin stabilisation in ASD [55]. This again is supported up by a particular enrichment for genes directly and indirectly associated with both cell adhesion and cytoskeletal associated cell membrane proteins in our interacting genes (5 out of 9; *discs large, p120 catenin, Notch, alpha-spectrin, pak*), several of which have been identified to have properties in the neuron [54,56–59]. Many studies have linked neurodevelopmental disorders, including ASD, to mutations in synaptic adhesion proteins, including the neurexins and neuroligins, and mutations in these in *Drosophila* have yielded both behavioural and larval NMJ defects [30,31,60]. We show specific interactions between *P120ctn, dlg* and *pak* with *Drosophila* neurexin IV, which has been shown to be involved in the maturation of the *Drosophila* NMJ. [34,61,62]. Notably, the ASD-network orthologues (namely *org-1, pasha* and *sep4*) that contribute to the interactions modelling the CNV 12239_chr22_loss_17249508_l that covers the 22q11.2 microdeletion critical region, did not yield phenotypes in the sensitised *NrxIV* background (Fig. 6) suggesting that these intracellular genes may be exerting phenotypic effects through an alternative process. While other (non-ASD network) genes in this 22q11.2 critical region have received interest in effecting the many associated phenotypes, our study suggests that interactions between the human genes *TBX1, DGCR8* and *SEPT5* may play a significant causal role [39].

Alterations in active zone structures have been connoted in ASD [63]. Moreover, neuron specific knockdown of the *Drosophila* orthologues of the ASD genes *CNTNAP2* and *NRXN1, NrxIX* and Nrx-1 (*dnrx*), have been shown to alter the levels of the active zone protein BRP [18]. BRP shows both sequence and functional homology with the mammalian ELKS/CAST proteins that are structural components of the vertebrate active zone [64,65]. Here we show that dosage changes created by transheretozygotes between *NrxIV, dlg* and *pak* lead to a reduction in BRP foci. Dlg is a postsynaptic anchoring protein which is required for the development and stability of the postsynaptic subsynaptic reticulum (SSR), whilst Pak is known to phosphorylate Dlg and control its abundance at the synapse [36]. *NrxIV* is predominantly presynaptic, but is required for the cell-cell contacts that influence synaptic development [66], and govern the interconnectivity between both neurons, glial cells and the pre- and postsynapse [30]. Dosage alterations in NrxIV with Dlg, Pak and p120 catenin may lead to alterations in adhesion protein interactions, causing the destabilisation of the synaptic architecture in both the pre- and postsynapse, ultimately leading to defective synaptic maturation. In the null mutant of the *Drosophila* orthologue of *NRXN1, Nrx-1* (*dnrx*), GluRIIA subunit fluorescence and BRP active zone density were increased, although bouton numbers still remain reduced [62]. It has been suggested that interactions between *Drosophila* neurexins and neuroligins may synchronise GluRIIA, and presynaptic active zone neurexin and neuroligin may be involved in the link between GluRIIA expression and presynaptic active zone dynamics [30,62]. The interactions observed between *P120ctn, NrxIV dlg* and *pak* also result in synaptic maturation defects. Null mutants in *pak* and *dlg* have also been shown to lead to alterations in glutamate receptor subunits (GluRIIA) [36], however, here we did not see a significant interaction between the *dlg/pak* transheterozygotes, or the interactions with *NrxIV*. GluRIIA levels were affected in the *pasha/Sep4* cross. Reductions in GluRIIA have been found to lead to a compensatory increase in active zone size [67]. We did not observe a change in active zone puncta in the *pasha/Sep4* cross, suggesting that these compensatory mechanisms may be compromised in this case. It is also worth noting that, through changes in the mammalian target of rapamycin mTOR, altered eIF4E-dependent translation results in ASD-relevant phenotypes in mouse [68] and altered regulation of the synthesis of neuroligins. Mutations in *Drosophila* TOR and eIF4E alter levels of GluRIIA but do not alter the active zones [69]. Interestingly, the fragile X syndrome associated protein FMRP (fragile X syndrome has 30% co-morbidity with ASD) and the miRNA pathway are known to mechanistically interact [70] (Pasha, is part of the miRNA microprocessor complex), while the mRNA of the *Sept4* human orthologue (*SEPT5*) is an FMRP target [10]. Both FMRP, which is known to pause ribosomal translocation [71], and Pasha are involved in translational repression [72,73]. In addition, both mutations in FMRP and the microRNA processing machinery affect the ratios of GluR subunits [43,74]. It may be that *pasha/Sep4* deficit leads to the suboptimal translation of *Sep4*, which functions in complexes that associate with cellular membranes and actin filaments. This may lead to inefficient synaptic anchoring. Further analysis of this process, and those arising from the gene-gene interactions in this study, can now be performed. In summary, our in vivo model system may be well suited to rapidly evaluate how combinations of genes may contribute synergistically to the neurological defects that, in turn, may contribute to ASD.

Although our data strongly supports a significant causal role for synergistic effects underlying ASD, our current study design is unable to reliably estimate the extent as it was limited to (*i*) considering only pairwise interactions among sets of candidate genes, defined as those genes whose protein products were identified as participating in an ASD-associated interaction network [9], (*ii*) considering a limited number of neurological phenotypes studied in the model organism *Drosophila* [11] and (*iii*) our study considered only those 4 multigenic *de novo* CNVs identified in individuals with ASD in previous studies where each candidate gene possessed a unique *Drosophila* orthologue (see **Methods**). Given that each CNV in those previous studies affected on average 16 protein-coding genes (including non-network genes), we might only expect only 4 genes to possess unique human:*Drosophila* orthologues (see **Methods**), severely limiting the ability of this model to examine all combinations of affected genes. However, given that even 16 genes per CNV would generate 240 pairwise gene combinations, it is difficult to imagine the extent and nature of these interactions being examined in a less tractable model with a higher ratio of unique orthologues. While we employed NMJ analysis as a tractable system for studying synaptic function, and circadian analysis to provide a high throughput method for studying behavioural deficit, it would be interesting to expand the behavioural assays to include those which studied social interaction, such as the social space index [75], and also courtship analysis [76]. Nonetheless, the relevance of our findings in *Drosophila* to humans is supported by the consistent directional effects observed between the increased or decreased bouton counts, which correspond well with the direction of gene dosage change in the human CNV. Taken together with the fact no non-ASD-associated network gene examined yielded abnormal phenotypic effects, when disrupted singularly or in combination (Figs. 2–5), the development of an informatics-targeted *Drosophila*-screen presents a rapid approach for identifying disease-relevant candidate interactions.

## Methods

### Selecting *Drosophila* orthologues of genes affected by *de novo* CNVs identified in individuals with autism

We considered the four sets of CNVs we informatically examined previously: (1) 73 *de novo* CNVs from the Autism Genome Project study [5], (2) 28 *de novo* CNVs from the Marshall *et al*. study [27], (3) 94 *de novo* or rare CNVs from the Levy *et al*. study[28] and (4) 67 *de novo* or rare CNVs from the Sanders *et al*. study [29]. On average each CNV overlaps 16 genes with an s.d. of 23 showing wide variation. In order to reduce the combinatorial search space, we considered only those 210 genes whose protein products had been identified in a previous CNV study to participate in a large and highly-significant network of interacting proteins with roles in neural functioning (herein termed the ASD-associated network) [9]. This network provides an aetiological basis through which genetic interactions might be mediated. We downloaded the set of the unique human:*Drosophila* orthologues as determined by the InParanoid tool [77]. Although our study has strictly focused on unique (1:1) orthologues, we note that a much larger number of *Drosophila* orthologues could be identified by relaxing the requirement of only a unique human orthologue [78]. Nonetheless, examining the 95 *de novo* CNVs that harboured genes from the ASD-associated network [9], we identified 7 CNVs for which a unique fly orthologue could be identified for every CNV-overlapped network gene (Table 1; Figs. 2–6, S1, S2). In addition, we selected two non-network genes from each CNV with multiple candidate genes, whose unique fly orthologues were neuronally-expressed in the larval stage. Acknowledging the limited number of unique human: *Drosophila* orthologues, we were not seeking here to exhaustively ascertain combinatorial effects in *Drosophila* between all simultaneously copy number changed genes in individuals with autism but rather to investigate the informatically-proposed presence of such effects *in vivo* (see Discussion). All selected genes were completely overlapped by their respective genes.

### *Drosophila* genetics

All *Drosophila* stocks were isogenised to the *w* ^*1118*^ wild type background for 7 generations. Where possible, previously described amorphic mutants were selected for analysis. Uncharacterised insertions were validated using deficiencies. Stocks were acquired for positive mutation hits from the Bloomington *Drosophila* Stock Center (BDSC, Indiana University) unless otherwise stated and contained the following insertions or lesions: *p120ctn* ^*308*^, *dlg* ^*1*^, *pak* ^*6*^, *hts* ^*k06121*^, *loco* ^*KG02176*^, *aux* ^*727*^, *org-1* ^*MB01466*^ and *pasha* ^*LL03360*^, α-*Spec* ^*rg41*^, *Sep4* ^*NP7170*^ (*Drosophila* Genetic Resource Center, Kyoto Institute of Technology), *CG13192* ^*EY07746*^, *CG34449* ^*d00976*^, *CtBP* ^*87De-10*^, CG8507^G4779^, htt^MB03997^. *w* ^*1118*^; *UAS-notch* ^*Full*^, UAS-alpha-spectrin and UAS-Dynactin (Shabire) were used for overexpression relating to gains. To generate these, the coding sequences were amplified using primers containing KpnI sites, subcloned into pUAST, and injected into embryos. The 1032-GAL4 ubiquitous driver was used for overexpression due to its moderate ubiquitous expression. *Nrx*IV^4304^ (BDSC, Indiana University) was used as a positive control. Negative controls were randomly selected from genes that were not picked as candidates from the CNV set. All negative controls selected displayed both larval and adult neuronal expression (BDSC, Indiana University). w^1118^; *Fsn* ^*KG08128*^, *w* ^*1118*^; *cg5359* ^*e03976*^, *w* ^*1118*^; *Hira* ^*185*^ *w* ^*1118*^; *sea* ^*EP3364*^, *w* ^*1118*^; *Su(P)* ^*EY13245*^, *w* ^*1118*^; *CG14104* ^*f07593*^, *w* ^*1118*^; *nelf-a* ^*KG09483*^, *w* ^*1118*^; *CG8507* ^*G4779*^ and *CG3321* ^*c00226*^ were used for negative controls.

### *Drosophila* larval NMJ analysis

All stocks were cultured on standard molasses/maize meal and agar medium in plastic vials or bottles at 25°C. Larvae were reared on apple juice plates supplemented with molasses/maize meal and yeast as previously described [79]. Larvae were selected for NMJ analysis at 5 days post egg laying. For analysis of bouton number was performed on the NMJ innervating muscles 6 and 7 from hemisegment A2 (1). Over 15 larvae were analysed for each genotype. For immunohistochemistry larvae were fixed for 20mins in 4% paraformaldehyde, or Bouin’s fixative for 30 minutes (GluRIIA). Primary antibodies used were anti-discs large (DLG, Developmental Studies Hybridoma Bank (DSHB), Iowa City, Iowa, USA),anti-Fas2 (DSHB), anti-GluRIIA (DSHB) and anti-BRP (DSHB), all used at 1/100. Secondary antibodies used were AlexaFluor 488 goat anti-rabbit and AlexaFluor 633 goat anti-mouse (Invitrogen) at 1/1000, and anti-HRP-TRITC (The Jackson Laboratory, Bar Harbor, Maine, USA). Z-stacks were taken using a laser-scanning confocal microscope (Leica TCS SP5 II confocal microscope) and analysis performed using ImageJ and Adobe Photoshop. For statistical analysis of the genetic interactions, ANOVA was performed between the control, the two single heterozygous mutations and the transheterozygotes.

### GluRIIA and BRP fluorescence analysis

For GluRIIA and BRP analysis at the NMJ, synapses were analysed with optical sections of 0.2μm using a laser-scanning confocal microscope (Leica TCS SP5 II confocal microscope) All digital analysis performed using ImageJ. For BRP staining the number of puntcta was scored over the synapse and normalized to synapse area. For GluRIIA analysis the average fluorescence intensity was analysed over the whole synapse (marked by HRP staining) and then normalized to HRP intensity. No alterations in HRP levels were observed in any genotypes.

### *Drosophila* sleep/wake circadian behavioural assays

All stocks and F1 crosses were cultured on standard molasses/maize meal and agar medium in plastic vials or bottles at 25°C within a light/dark cycle of 12 hrs light/ 12 hrs dark (12:12 LD). For overexpressions, flies were reared at 16°C and then switched during late pupation so to mitigate gross developmental defects. The flies were then transferred to 25°C within a light/dark cycle of 12 hrs light/12 hrs dark (12:12 LD). Flies selected for analysis were between 3 and 5 days old. Flies were the transferred to activity tubes containing 5% sucrose and 2% Bacto agar at one end and were continually synchronized and entrained using a light/dark cycle of 12 hrs light/12 hrs dark (12:12 LD) at 25°C in the circadian incubator for 3 days before data collection. The flies were then switched analysed for experimentation and data collection. Sleep/rest periods were identified as contiguous 5 minute periods of inactivity and were scored and averaged over 2 day period for both dark ‘day’ and ‘night’ cycles. The raw binary data is processed using DAM Filescan102X (Trikinetics, Inc.) and summed into 5 minute bins when analysing sleep/rest parameters. Data analysis was performed within Excel. Statistics were performed using student’s t-tests between ‘day’ and ‘night’ activity.

## Supporting Information

S1 Fig No significant interactions were observed between the pairwise crosses of *Drosophila* gene orthologues associated with the human de novo loss _l 12691.p1_chr16_loss_68529466_s. A) NMJ morphology and B) circadian analysis for *Drosophila* orthologues of ASD candidate genes (Table 1). No NMJ or light/dark bias changes were observed in any of the single heterozygous mutants or pairwise crosses.
(TIF)

S2 Fig The interactions observed between the *Drosophila* orthologues of the human CNVs candidates (bold) and control genes. Green indicates an interaction, red no interaction.
(TIF)
