# Supplementary figures and images for: Synergistic Interactions between Drosophila Orthologues of Genes Spanned by De Novo Human CNVs Support Multiple-Hit Models of Autism
Source: PLoS Genet. 2015 Mar 27;11(3):e1004998. doi: 10.1371/journal.pgen.1004998 (PMC4376901; doi:10.1371/journal.pgen.1004998)

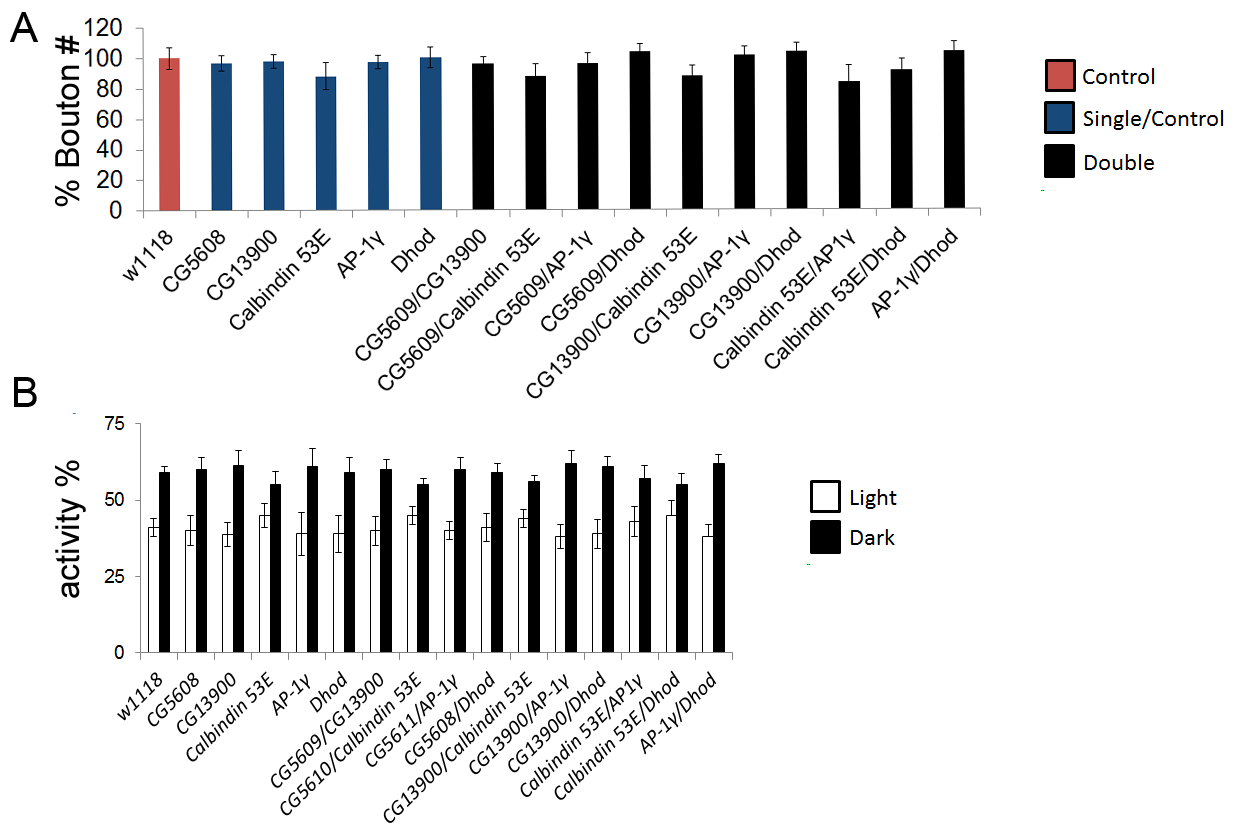

Supplement: S1 Fig — A) NMJ morphology and B) circadian analysis for Drosophila orthologues of ASD candidate genes (Table 1). No NMJ or light/dark bias changes were observed in any of the single heterozygous mutants or pairwise crosses. (TIF) [file pgen.1004998.s001.tif]

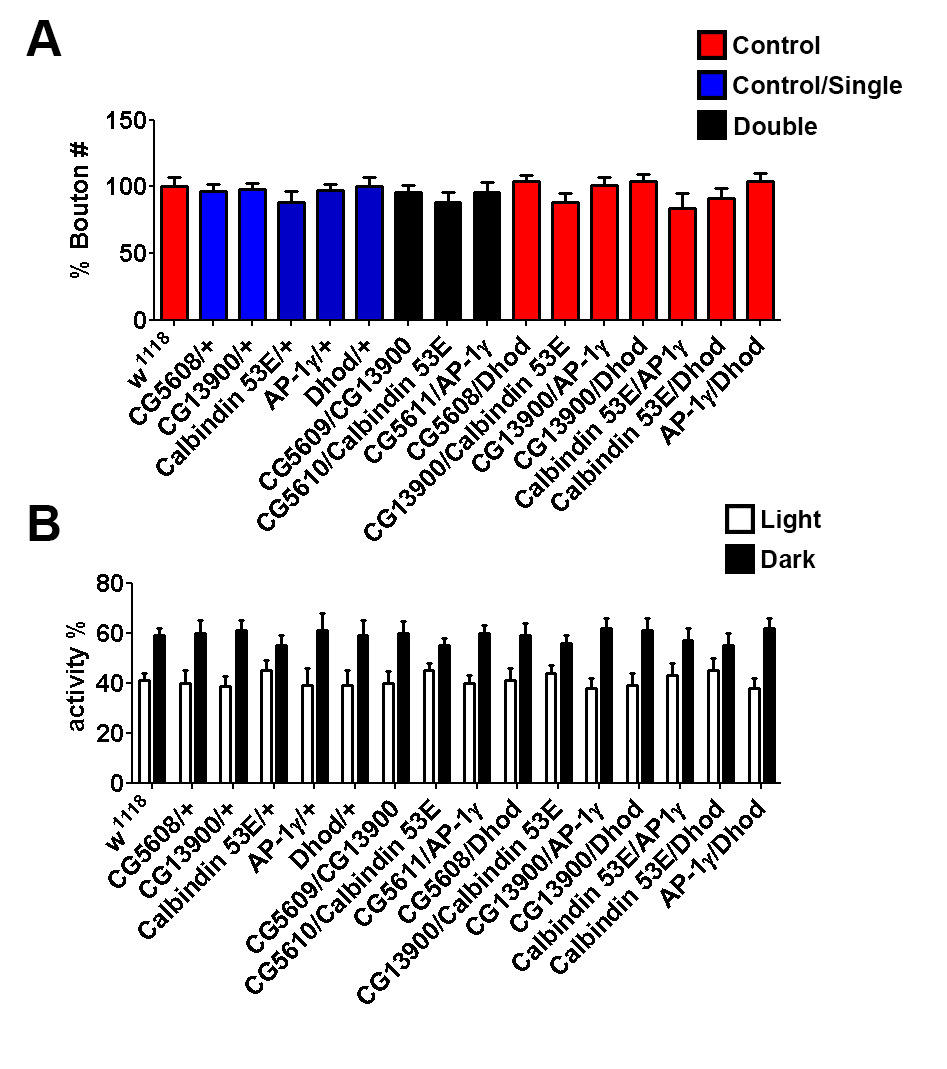

Supplement: S2 Fig — Green indicates an interaction, red no interaction. (TIF) [file pgen.1004998.s002.tif]
